# Verruciform xanthoma of the glans penis in a male with undiagnosed genital lichen sclerosus

**DOI:** 10.1002/ski2.375

**Published:** 2024-03-21

**Authors:** David J. Chandler, Dong Eun Lee, Mara Quante, Lindsay Atkinson

**Affiliations:** ^1^ Dermatology Department Brighton General Hospital University Hospitals Sussex NHS Foundation Trust Brighton UK; ^2^ Department of Global Health and Infection Brighton and Sussex Medical School Brighton UK; ^3^ Histopathology Department Royal Sussex County Hospital University Hospitals Sussex NHS Foundation Trust Brighton UK

## Abstract

We report the second case of verruciform xanthoma of the penis occurring in a male with genital lichen sclerosus. We suggest that the diagnosis of VEGAS xanthoma should prompt careful examination of the genitalia for evidence of underlying lichen sclerosus, the signs of which may be subtle.

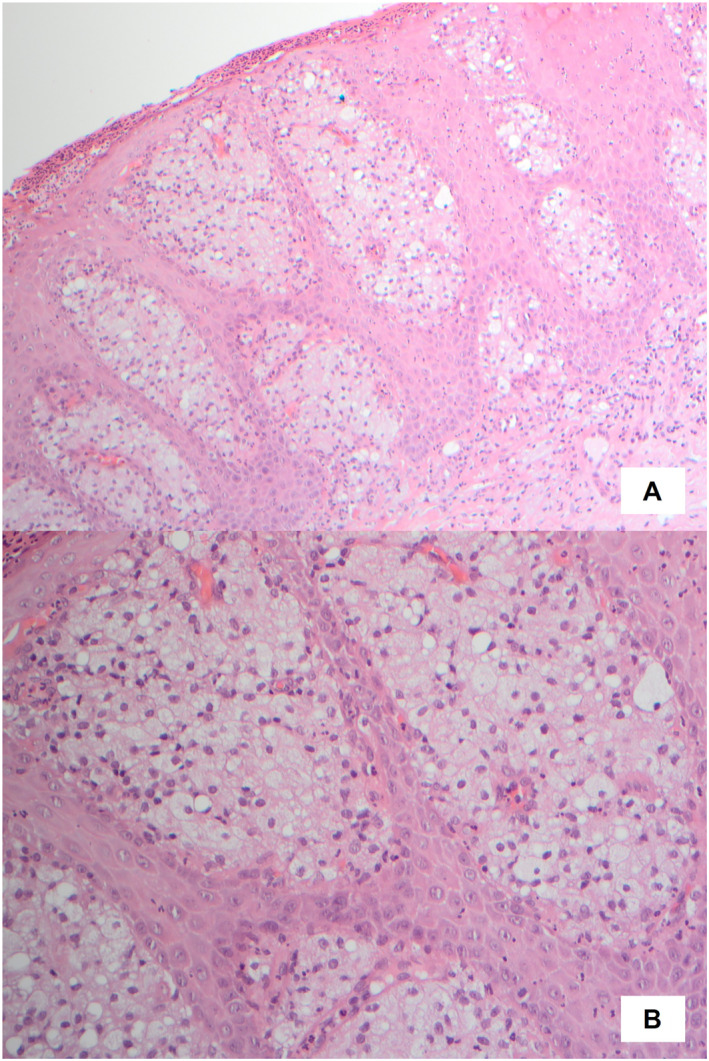

Dear Editor, A 39 year old male presented with a 4 weeks history of an asymptomatic lesion on the glans penis. A superficial shave biopsy performed 2 weeks earlier had shown a hypertrophic epithelium with prominent parakeratosis suggestive of condyloma acuminatum.

His medical history was significant for asthma, previous episodes of cholecystitis and pilonidal sinus. There was no past history of skin disease or sexually‐transmitted infection and he denied any genitourinary symptoms. His regular medications included montelukast sodium and inhaled budesonide/formoterol (DuoResp Spiromax).

Examination revealed a 1 cm white‐yellow warty plaque on the glans penis (Figure 1). Chronic changes of lichen sclerosus were also noted. These included etiolation of the glans penis, loss of architectural definition posteriorly with complete loss of the frenulum, and constrictive lichenoid posthitis; although he was able to fully retract the foreskin. The patient reported being aware of some of these changes since childhood, however he was unaware that they were abnormal. There were no palpable lymph nodes.

**FIGURE 1:**
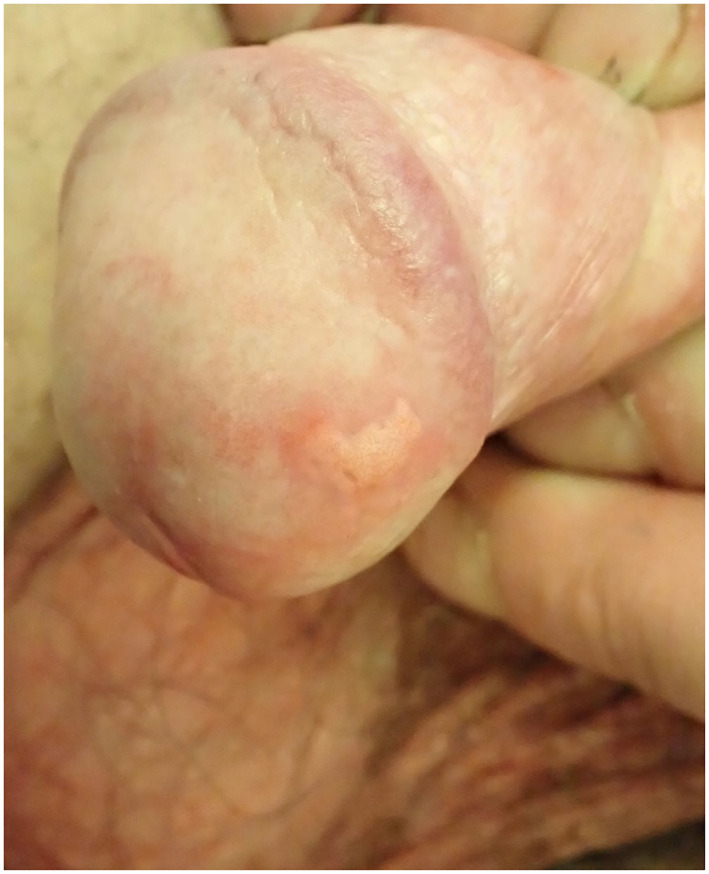
Clinical appearance of verruciform xanthoma. A yellowish cerebriform plaque on the glans penis.

Clobetasol ointment was commenced once daily, however after 4 weeks there was no significant improvement in the lesion and a punch biopsy was performed. This revealed a slightly acanthotic epidermis with superficial erosions and associated neutrophils (Figure 2a). Within the papillary dermis, large foamy histiocytes were seen with associated lymphoplasmacytic inflammation (Figure 2a,b). There was no dysplasia or malignancy, and no fungal organisms were identified.

**FIGURE 2:**
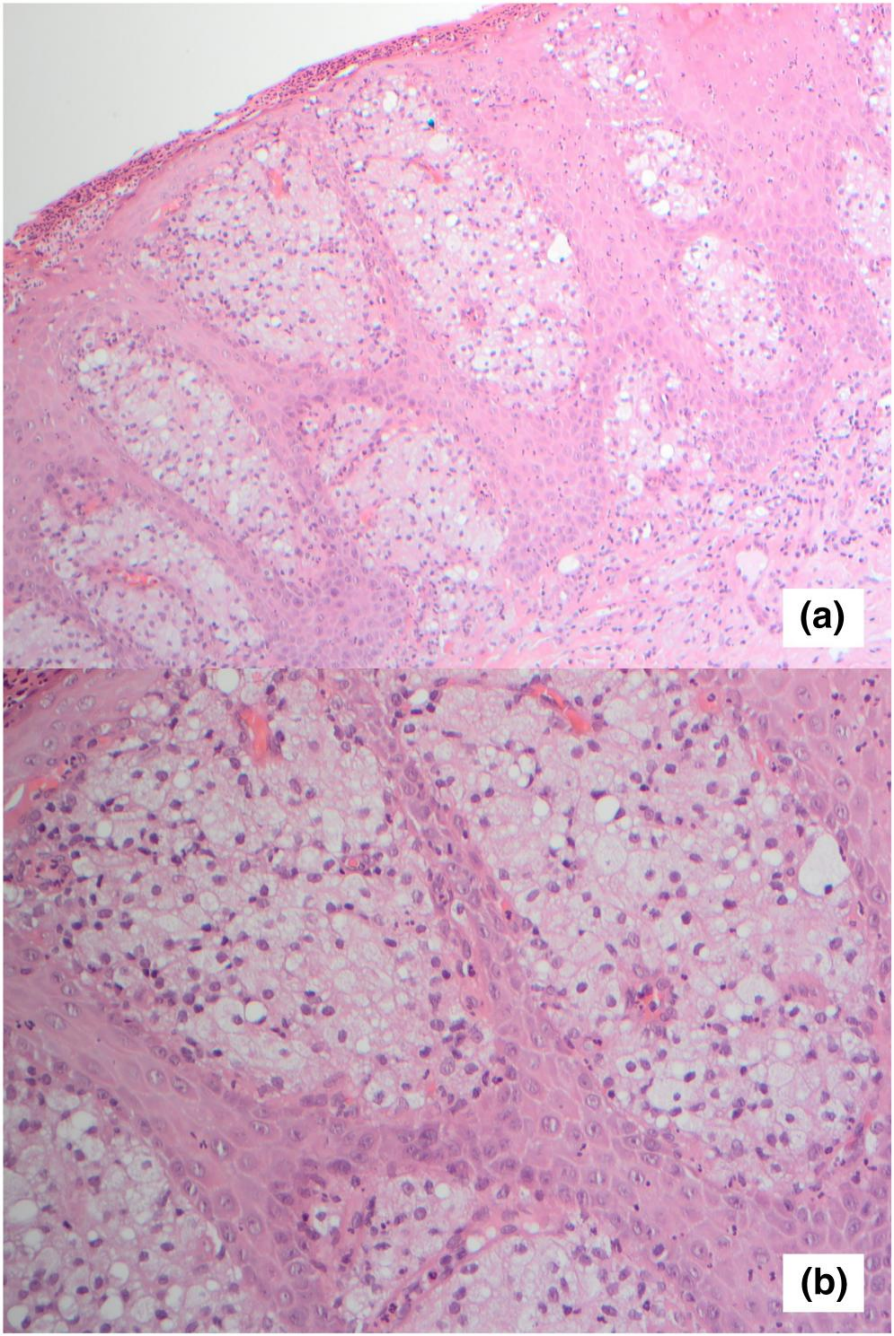
(a and b) Histological appearance of verruciform xanthoma. Large foamy histiocytes are seen within the papillary dermis.

The clinical and histological features together confirmed the diagnosis of verruciform xanthoma.

Verruciform xanthoma is a benign warty lesion most commonly arising in the oral cavity. Lesions are usually asymptomatic and have variable morphology ranging from flat to papillary and verrucous. Extraoral lesions are uncommon but have been described affecting both male and female genitalia where they are referred to as verruciform genital‐associated (VEGAS) xanthomas. Other extraoral sites include the face, limbs, and acral skin. Lesions can be multiple.

Verruciform xanthoma of the penis is rare. The first case was reported by Kraemer et al in 1981^1^ and to date a total of 41 cases have been reported in the literature. The age of onset ranges from 8 to 85 years, and the average duration of symptoms prior to diagnosis is 3.7 years^2^ Lesions are typically solitary and enlarge slowly. The most common location is the glans followed by the prepuce, coronal sulcus and shaft of the penis.^2^ Lesions vary from sessile to pedunculated, and are often pink or skin‐coloured to yellow. Some lesions show porokeratosis‐like features clinically and histologically.

Verruciform xanthoma may resemble condyloma acuminatum, however the latter frequently presents with multiple lesions whilst verruciform xanthoma is usually solitary. Squamous cell carcinoma and verrucous carcinoma must be excluded. Other differential diagnoses to consider include bowenoid papulosis, verruca vulgaris, granular cell tumour, giant molluscum contagiousm, condyloma latum, and seborrhoeic keratosis. A biopsy is essential to confirm the diagnosis. The histological features of verruciform xanthoma include acanthosis with parakeratosis, uniformly elongated rete ridges, neutrophilic dermal infiltrate, and the presence of foamy histiocytes within the dermal papillae. Foam cells stain positive for Periodic Acid Schiff and CD68.

Treatment of verruciform xanthoma may not be required beyond reassurance. Surgical excision is curative. Other treatment modalities which have been successful include carbon dioxide laser, superficial electrocoagulation, cryotherapy, imiquimod and radiotherapy.^3^

The aetiology of verruciform xanthoma is poorly understood. An association with human papilloma virus (HPV) infection has been suggested, however the absence of histological features of HPV infection and the failure to detect HPV DNA in numerous cases of verruciform xanthoma is not in favour of this. One hypothesis is that verruciform xanthoma represents a reaction to epithelial damage arising from local trauma or inflammation. This is supported by reports of verruciform xanthoma occurring in the setting of congenital hemidysplasia with ichthyosiform erythroderma and limb defects syndrome, inflammatory linear verrucous epidermal nevus, graft versus host disease, discoid lupus erythematosus, pemphigus vulgaris, and recessive dystrophic epidermolysis bullosa. The proposed mechanism involves entrapment and degradation of keratinocytes in the papillary dermis with release of lipids which are engulfed by macrophages.

A similar inflammatory reaction, involving degeneration of keratinocytes and the dermoepidermal junction, occurs in lichen planus and lichen sclerosus. VEGAS xanthomas have been reported previously in association with genital lichen planus and lichen sclerosus in females,^4^ and one case has been reported in a male patient with genital lichen sclerosus.^5^

To our knowledge, this is the second report of verruciform xanthoma of the penis occurring in a male with genital lichen sclerosus. We theorize that this could have occurred secondary to the effects of chronic local inflammation from untreated lichen sclerosus. Altered lymphatic drainage and lymphostasis has been proposed as a contributing factor in the pathogenesis of verruciform xanthoma, in patients with lichen sclerosus.^6^ We suggest that the diagnosis of VEGAS xanthoma should prompt careful examination of the genitalia for evidence of an underlying inflammatory dermatosis such as lichen sclerosus, the signs of which may be subtle.

## CONFLICT OF INTEREST STATEMENT

None to declare.

## AUTHOR CONTRIBUTIONS

**David J. Chandler**: Conceptualization (lead); investigation (lead); writing – original draft (lead); writing – review & editing (supporting). **Dong Eun Lee**: Investigation (supporting); writing – original draft (supporting). **Mara Quante**: Formal analysis (lead); writing – review & editing (supporting). **Lindsay Atkinson**: Investigation (supporting); supervision (lead); writing – review & editing (lead).

## FUNDING INFORMATION

This article received no specific grant from any funding agency in the public, commercial, or not‐for‐profit sectors.

## ETHICS STATEMENT

Not applicable.

## Data Availability

The data underlying this article will be shared on reasonable request to the corresponding author.

## References

[1] Kraemer BB , Schmidt WA , Foucar E , Rosen T . Verruciform xanthoma of the penis. Arch Dermatol [Internet]. 1981;117(8):516–518. 10.1001/archderm.117.8.516 7259249

[2] Stiff KM , Cohen PR . Vegas (verruciform genital‐associated) xanthoma: a comprehensive literature review. Dermatol Ther [Internet]. 2017;7(1):65–79. 10.1007/s13555-016-0155-0 PMC5336425 27848170

[3] Sette CS , Wachholz PA , Brandão LSG , Marques GF , Casafus FS , Soares CT . Verruciform xanthoma on the penis: an unusual location. Clin Exp Dermatol [Internet]. 2015;40(7):807–808. 10.1111/ced.12579 25787016

[4] Fite C , Plantier F , Dupin N , Avril M‐F , Moyal‐Barracco M . Vulvar verruciform xanthoma: ten cases associated with lichen sclerosus, lichen planus, or other conditions. Arch Dermatol [Internet]. 2011;147(9):1087–1092. 10.1001/archdermatol.2011.113 21576553

[5] Ong ELH , Ratynska M , Sherring K , Bunker C . An unusual penile lesion. Clin Exp Dermatol [Internet]. 2022;47(12):2314–2317. 10.1111/ced.15329 36052746

[6] Carlson JA , Carlson GDD , Murphy M , Rohwedder A . Lichen sclerosus exhibiting histologic signs of lymphedema: an essential factor in the pathogenesis of verruciform xanthoma. Arch Dermatol [Internet]. 2012;148(2):260–262; author reply 262. 10.1001/archdermatol.2011.1536 22351836

